# Proliferating macrophages in human tumours show characteristics of monocytes responding to myelopoietic growth factors

**DOI:** 10.3389/fimmu.2024.1412076

**Published:** 2024-06-05

**Authors:** Saem Mul Park, Chun-Jen J. Chen, Daniel J. Verdon, Marcus P. Y. Ooi, Anna E. S. Brooks, Richard C. W. Martin, Jon A. Mathy, Patrick O. Emanuel, P. Rod Dunbar

**Affiliations:** ^1^ School of Biological Sciences, The University of Auckland, Auckland, New Zealand; ^2^ Maurice Wilkins Centre, Auckland, New Zealand; ^3^ Department of Surgery, Te Whatu Ora Waitemata, Auckland, New Zealand; ^4^ Department of Surgery, Faculty of Medical Health Sciences, The University of Auckland, Auckland, New Zealand; ^5^ Auckland Regional Plastic, Reconstructive and Hand Surgery Unit, Auckland, New Zealand; ^6^ Department of Dermatology, Icahn School of Medicine at Mount Sinai, New York, NY, United States

**Keywords:** tumour-associated macrophages, macrophage proliferation, immunosuppression, melanoma, tumour microenvironment

## Abstract

Macrophages play essential roles in maintaining tissue homeostasis and immune defence. However, their extensive infiltration into tumours has been linked to adverse outcomes in multiple human cancers. Within the tumour microenvironment (TME), tumour-associated macrophages (TAMs) promote tumour growth and metastasis, making them prime targets for cancer immunotherapy. Recent single-cell analysis suggest that proliferating TAMs accumulate in human cancers, yet their origins and differentiation pathways remain uncertain. Here, we show that a subpopulation of CD163+ TAMs proliferates *in situ* within the TME of melanoma, lung cancer, and breast cancer. Consistent with their potential role in suppressing anti-tumour activities of T cells, CD163+ TAMs express a range of potent immunosuppressive molecules, including PD-L1, PD-L2, IL-10, and TGF-β. Other phenotypic markers strongly suggested that these cells originate from CD14+ CCR2+ monocytes, a cell population believed to have minimal capacity for proliferation. However, we demonstrate *in vitro* that certain myelopoietic cytokines commonly available within the TME induce robust proliferation of human monocytes, especially the combination of interleukin 3 (IL-3) and Macrophage Colony-Stimulating Factor 1 (M-CSF). Monocytic cells cultured with these cytokines efficiently modulate T cell proliferation, and their molecular phenotype recapitulates that of CD163+ TAMs. IL-3-driven proliferation of monocytic cells can be completely blocked by IL-4, associated with the induction of CDKN1A, alongside the upregulation of transcription factors linked to dendritic cell function, such as BATF3 and IRF4. Taken together, our work suggests several novel therapeutic routes to reducing immunosuppressive TAMs in human tumours, from blocking chemokine-mediated recruitment of monocytes to blocking their proliferation.

## Introduction

Tumour growth is associated with the accumulation of various myeloid cell types. In particular, tumour-associated macrophages (TAMs) represent one of the most abundant immune cell types in tumours, and their infiltration is associated with poor prognosis in most cancers (1). TAMs can promote cancer progression by stimulating angiogenesis, increasing tumour cell survival, migration, and invasion, and suppressing anti-tumour immune responses. TAMs can also interfere with the efficacy of T cell-based checkpoint blockade, and therefore represent a major target for cancer immunotherapy (1). TAMs are highly heterogeneous and comprise various subpopulations with unique transcriptional profiles (2). Thus, developing more effective therapeutic strategies targeting TAMs requires a better understanding of the diversity of TAMs and their development pathways.

CD163+ TAMs are known to be strongly associated with poor outcomes in several human tumour types (3), including melanoma (4). In an experimental melanoma model resistant to anti-PD-1 immunotherapy, CD163+ TAMs maintain immune suppression, and specific targeting of these TAMs restores anti-tumour T cell responses (5). CD163 is often used as a marker of the so-called “M2” or “alternatively activated” macrophages that are thought to be polarised by Th2-derived cytokines (e.g. IL-4, IL-10, IL-13, TGF-β and PGE2) and promote tissue repair via tissue remodelling and immune modulation (6). However, increasing evidence now indicates that TAMs exhibit multiple distinct phenotypic states, as evidenced by their different transcriptional states, and that the paradigm of a switch between M1 and M2 states is an over-simplification of these states in human tumours (2, 7).

Despite their significant roles in promoting tumour progression to malignancy, the origin of TAMs in human cancer is still incompletely understood. Studies using animal models showed that TAMs originate from both bone marrow-derived monocytes and tissue-resident macrophage progenitors, and the signals derived from the tumour microenvironment (TME) differentiate them into pro-tumoural phenotypes (1, 8–10). Some animal models also demonstrated TAMs proliferate within tumours (8, 11). The presence of proliferating macrophages in human cancer has occasionally been noted (12), although their origin has not been clearly addressed.

Here we initially sought to examine the origin of CD163+ TAMs accumulated in human metastatic melanoma. Our results show that CD163+ TAMs display the molecular signatures originating from blood monocytes and express a range of molecules known to suppress T cell functions. Interestingly, many CD163+ TAMs were found to proliferate within the TME of various cancers. We hypothesised that these cells proliferate as they differentiate from monocytic precursors – even though circulating human monocytes are typically regarded as having limited proliferative capacity. As a proof of concept, we then demonstrated that we could generate proliferating macrophages *in vitro* from human blood monocytes that recapitulate the phenotype of TAMs. These *in vitro* generated TAMs efficiently suppressed T cell functions, and IL-4 could block their proliferation. Collectively, our results have implications for developing cancer therapies targeting TAMs.

## Materials and methods

### Tissue samples

Fresh-frozen or formalin-fixed and paraffin-embedded (FFPE) metastatic melanoma tumour specimens and lung cancer and breast cancer tumour specimens were obtained from patients undergoing excisional surgery. Clinical details of the tissue samples examined in this study are available in **Supplementary Table 1** and **2**. Written informed consent was obtained under protocols approved by the Northern Regional Ethics Committee, New Zealand.

### Antibodies and reagents

Antibodies (Abs) used for multicolour immunofluorescence microscopy and flow cytometry are detailed in **Supplementary Table 3**. Unconjugated primary Abs were detected using isotype-specific goat anti-mouse or anti-rabbit Abs conjugated to Alexa Fluor 488, 555 or 647 (Invitrogen).

### Multicolour immunofluorescence microscopy

Fresh-frozen tissue sections were fixed with acetone. FFPE tissue sections were dewaxed in xylene, rehydrated through graded concentrations of ethanol, and subjected to heat-mediated antigen retrieval using the R-buffer A (EMS) or Tris-EDTA buffer (abcam). Tissue sections were blocked with 0.25% casein and 10% human serum, probed with primary Abs (**Supplementary Table 2**), and then with secondary Abs. For immunocytochemistry, *in-vitro* cultured cells were fixed with 70% ethanol, blocked with 10% human serum, and probed with primary and secondary Abs. All tissue and cell samples were counterstained with DAPI, mounted using Prolong Gold (Invitrogen), and visualised with an Eclipse Nikon fluorescent microscope (Nikon) equipped with the epi-fluorescent filters: UV, 450-490nm, 530-560nm and 590-650 nm. Images were generated using Cytosketch (CytoCode).

### Fluorescent *in situ* hybridisation

Detection of mRNA on tissue sections was performed using the RNAscope assay kit (ACD) and human CCL2, CX3CL1, TGF-β, IL-10 and M-CSF probes (ACD) according to the manufacturer’s instruction. Briefly, tissue sections were fixed with 4% paraformaldehyde, dehydrated, and then treated with Protease IV. Following the hybridisation at 40°C, sections were counterstained with anti-CD163 and DAPI. DapB probe was used as a negative control.

### Cell isolation, culture, and flow cytometry

Human PBMCs were obtained from healthy donors by gradient separation using Lymphoprep™ (Alere Technologies). From PBMCs, T cells were isolated using the Pan T Isolation Kit II (Miltenyi Biotec), and monocytes were isolated using the Monocyte Isolation Kit II or CD14 microbeads (Miltenyi Biotec). To assess cell proliferation, isolated cells were first labelled with the CellTrace™ Violet (CTV) dye (Invitrogen) before culture. Monocytes were cultured in a U-bottom plate containing CellGenix^R^ GMP DC medium (GellGenix) supplemented with 1% human serum and different combinations of the cytokines, including FLT3L (100 ng/ml, PeproTech), IL-3 (5 or 10 ng/ml, PeproTech), IL-4 (50 ng/ml, PeproTech), GM-CSF (100 ng/ml, PeproTech), and M-CSF (100 ng/ml, PeproTech). Subsequently, cultured cells were stained with fluorophore-conjugated Abs (**Supplementary Table 2**), and Propidium Iodide, and their proliferation and phenotype were measured by flow cytometry using a BD FACSAria™ II. Flow cytometry data were analysed using FlowJo V10.8.1 (Treestar).

### Gene expression analysis

“moDCs” cultured with IL-4 and GM-CSF and “moMACs” cultured with M-CSF were collected on day 5. “moTAMs” generated with IL-3 and M-CSF were divided into proliferating and non-proliferating fractions by cell sorting on day 7. RNA was extracted from collected cells using the RNeasy mini kit (QIAGEN). Gene expression profiles of different monocytic cell subtypes were assessed using the nCounter^®^ Human Myeloid Panel (NanoString). Pathway scores were generated by nSolver advanced analysis module (NanoString).

### T cell assays

A mixed leukocyte reaction was used to assess the stimulation of allogenic T cells by different monocytic cell populations. CTV-labelled T cells were co-cultured with unlabelled monocytic cells generated from a different donor in RPMI medium supplemented with 5% human serum, IL-2 (10 ng/ml, PeproTech) and IL-7 (10 ng/ml, PeproTech) for 6 days. To assess the ability of different monocytic cell types to inhibit antigen-specific expansion of a CD8+ T cell clone specific for the MART1/Melan A-derived peptide EAAGIGLTV, PBMCs were first pulsed with the potent analogue peptide ELAGIGLTV (2.5 µM) and then washed three times to remove any remaining unbound peptides. Subsequently, CTV-labelled clonal T cells were combined with peptide-loaded PBMCs at a 1:2 ratio, then transferred into 96-well plate wells containing 5x10^4^ phenotypically polarised monocytic populations. All cells were co-cultured in RPMI medium supplemented with 5% human serum, IL-2, and IL-7 for 6 days. The percentage of divided T cells and visualisation of cell division peaks were analysed by flow cytometry.

### Statistical analysis

All statistical and graphical analyses were performed using GraphPad Prism Software (V9). A one-way analysis of variance (ANOVA) with Tukey’s or Dunnett’s multiple comparisons test was used to assess statistically significant differences. P-values less than 0.05 were considered significant.

## Results

### CD163+ TAMs express a range of immunosuppressive molecules

CD163 is a widely used marker to locate human TAMs (3). CD163 expression has prognostic significance and correlates with lower overall survival in multiple human cancers, including melanoma (4, 13). However, functional properties of the CD163+ TAMs within the TME remain incompletely understood. We therefore sought to examine molecular profiles of CD163+ TAMs in the melanoma TME using multicolour immunofluorescence microscopy, especially their expression of the molecules involved in immune suppression.

**Figure 1:**
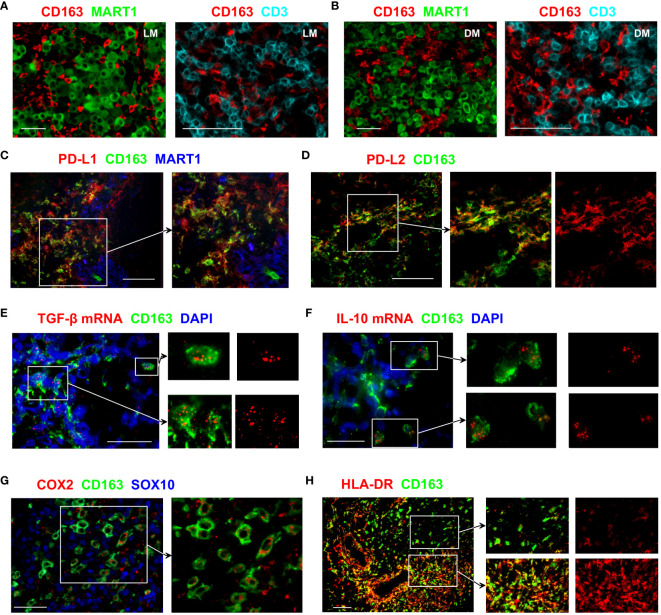
Immunosuppressive features of CD163+ TAMs. **(A, B)** Tissue sections from LN **(A)** or dermal **(B)** melanoma metastasis were probed with antibodies against CD163, MART1/Melan A and CD3 to assess the distribution of CD163+ TAMs, T cells and melanoma tumour cells. **(C–H)** Tissue sections from metastatic melanoma were stained with antibodies against the indicated markers to examine the functional phenotype of CD163+ TAMs. Images show that subsets of CD163+ TAMs express PD-L1, PD-L2, TGF-β, IL-10, COX2 and HLA-DR. Expression of MART1/Melan A and SOX10 shows the location of melanoma cells. In **(E, F)**, transcripts of TGF-β and IL-10 were detected using fluorescent *in situ* hybridisation. DAPI was used as a nuclear stain. Data shown are representative of at least 3 to 10 different metastatic melanoma cases. Scale bars represent 50 µm **(A, B, E, F, H)** or 100 µm **(C, D, G)**. Magnification: x10 (**A, B** left, **H**), x20 (**A, B** right, **C–E, G**) and x40 **(F)**. LN metastasis. DM, dermal metastasis.

CD163+ TAMs frequently infiltrated lymph node (LN) and dermal metastases, where they closely interacted with both melanoma tumours and T cells (**Figures 1A, B**). Programmed death-ligand 1 (PD-L1), an immune checkpoint protein that mediates immune escape in the TME by suppressing T cell activities (14), is known to be expressed by some immune cells as well as cancer cells. In the melanoma samples we examined, numerous CD163+ TAMs co-expressed PD-L1 (**Figure 1C**). We also noted PD-L1 expression in CD163− MART1/Melan A− cells that are likely to indicate the expression by other tumour-infiltrating myeloid cell types. In contrast, most melanoma cells lacked PD-L1 expression (**Figure 1C**). Some CD163+ TAMs also expressed PD-L2 (**Figure 1D**), a second ligand for PD-1 known to inhibit T cell activation (15).

Transforming growth factor-beta 1 (TGF-β1) is one of the key cytokines involved in promoting tumour growth, angiogenesis, and immunosuppression (16). Many CD163+ TAMs showed strong TGF-β1 mRNA signals, and positive expression was also noted in other cells that are CD163− (**Figure 1E**). IL-10 is a cytokine traditionally considered immunosuppressive due to its anti-inflammatory effects and capacity to inhibit T cell activation (17). Occasional CD163+ TAMs that strongly express IL-10 transcripts were observed in the melanoma TME (**Figure 1F**), although the vast majority of CD163+ TAMs were negative for IL-10 mRNA.

Expression of cyclooxygenase-2 (COX-2), an enzyme involved in the synthesis of prostaglandin E2 (PGE2), contributes to tumour immune evasion by regulating T cell infiltration and their functions (18). In melanoma, COX-2 expression correlates with a depth of tumour invasion and frequency of LN involvement (4). In the LN metastasis samples we examined, positive COX-2 expression was observed in a subset of the CD163+ TAMs, while the expression was often absent in the neighbouring melanoma cells (**Figure 1G**).

Expression of HLA-DR is used to identify CD14+ HLA-DR^lo/neg^ myeloid-derived suppressor cells (MDSC) that negatively correlate with responses to cancer immunotherapy (19). CD163+ TAMs in the melanoma TME showed highly variable levels of HLA-DR expression. Some CD163+ TAMs had low HLA-DR expression, whereas other CD163+ TAMs, especially those close to the vascular structures, brightly expressed HLA-DR (**Figure 1H**). This expression pattern is likely to indicate the downregulation of HLA-DR on some CD163+ TAMs that may relate to immunosuppressive function, similar to that reported for MDSC. Collectively, these results support the notion that CD163+ TAMs contribute to tumour progression.

### A subset of CD163+ TAMs originates from blood monocytes and locally proliferates within human tumours

**Figure 2:**
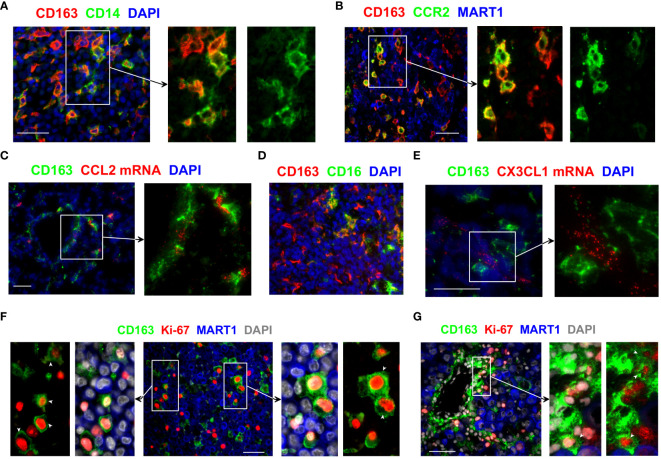
Monocytic origin of CD163+ TAMs and their proliferation within the melanoma TME. **(A, B, D)** The phenotype of CD163+ TAMs was assessed by co-staining with antibodies against CD14, CCR2 and CD16. **(C, E)** Tissue sections were examined for CCL2 and CX3CL1 mRNA using fluorescent *in situ* hybridisation and subsequently stained anti-CD163 to locate CD163+ TAMs. **(F, G)** Tissue sections from metastatic melanoma were probed with antibodies against CD163 and Ki-67 to assess the proliferation of CD163+ TAMs. MART1/Melan A expression was used to locate melanoma cells. DAPI was used as a nuclear stain. Results shown are representative of at least 3 to 15 different metastatic melanoma cases. Scale bars represent 50 µm. Magnification: x20 **(A–D)** and x40 **(E–G)**.

We then examined how CD163+ TAMs are likely to accumulate within the TME. CD163+ TAMs in the melanoma TME displayed molecular features suggesting their monocytic origin. In particular, a subset of CD163+ TAMs co-expressed variable levels of CD14 and CCR2 (**Figures 2A, B**), the key markers of the classical CD16− monocytes (20). While some CD163+ TAMs highly expressed CD14, many CD163 ^bright^ TAMs expressed only low or undetectable levels of CD14 (**Figure 2A**). Similarly, some CD163+ TAMs showed bright CCR2 expression, whereas other closely located CD163+ TAMs were either CCR2 negative or dim (**Figure 2B**). Consistent with the recruitment of the CCR2+ monocytes, we detected the presence of the CCR2 ligand CCL2 in the vicinity of CD163+ TAMs (**Figure 2C**).

These results show the phenotypic heterogeneity within CD163+ TAMs and confirm that at least a subset of CD163+ TAMs derive from CD14+ monocytes. We postulate that the classical CD16− monocytes are recruited into the melanoma TME, likely via tumour-derived CCL2, where they upregulate CD163 and gradually lose the expression of CD14 and CCR2. Of note, we also observed some CD163+ TAMs that co-expressed CD16 (**Figure 2D**). While this suggests that some of these recruited monocytes may gain CD16 expression as they differentiate within tumours, we cannot rule out the possibility of the direct recruitment of the non-classical CD16+ monocytes into the tumours, likely via CX3CL1 available in the melanoma TME (**Figure 2E**).

We hypothesised that not only the recruitment of monocytes but also the local proliferation of CD163+ TAMs and/or their monocytic precursors within the TME might contribute to the accumulation of these TAMs in human tumours. We found that a subset of CD163+ TAMs in metastatic melanoma co-expressed the cell proliferation marker Ki-67 (**Figures 2F, G**, **Supplementary Figure 1A**), indicating these TAMs indeed proliferate within the melanoma TME. Some of the Ki-67+ CD163+ TAMs were found in the vicinity of blood vessels, closely associated with neighbouring tumour cells (**Figure 2G**). While their frequencies varied between samples, proliferating CD163+ TAMs were found in most of the metastatic melanoma samples we examined. We also observed the presence of Ki-67+ CD163+ TAMs in tumours from patients with primary and metastatic breast cancer and lung cancer (**Supplementary Figures 1B, C**), suggesting proliferating TAMs contribute to the accumulation of TAMs in many cancer types.

### Myelopoietic growth factors induce robust proliferation of human monocytes

Our data raised the possibility that CD163+ TAMs that originate from blood monocytes expand within tumours in response to signals within the TME. We therefore tested whether we could generate cells that resemble CD163+ TAMs from monocytes *in vitro* and induce their proliferation with any factors likely to be available within the TME. Several hematopoietic cytokines have been reported to induce human monocytes to enter the cell cycle (20–26). However, there is currently a lack of evidence demonstrating that human monocytes can undergo multiple cell divisions under the influence of these cytokines.

**Figure 3:**
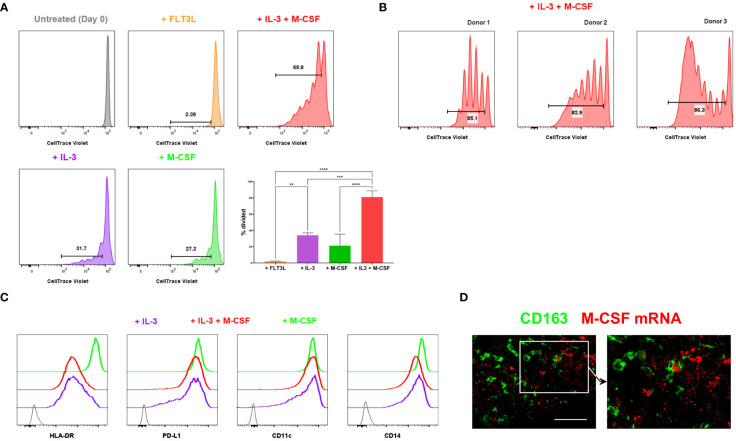
Proliferation of monocytic cells induced by myelopoietic growth factors. **(A, B)** Monocytes isolated from human blood were CTV-labelled and cultured with the indicated cytokines. *In vitro* proliferation of monocytic cells was assessed by flow cytometry. The depicted cells were gated on live, CD14+ HLA-DR+ monocytic cells. The percentages of divided cells are shown. Bar graphs shown in **(A)** are from the combined data from 3 independent experiments. Plots in **(B)** show the donor-to-donor variability in the proliferation of monocytic cells induced by IL-3 and M-CSF. **(C)** Monocytic cells cultured in varying cytokine conditions were examined for their cell surface expression of HLA-DR, PD-L1, CD11c and CD14 using flow cytometry. Grey indicates unstained negative control. Data shown in **(A–C)** are representative of at least 3 independent experiments using different biological replicates. **(D)** M-CSF mRNA was detected using fluorescent *in situ* hybridisation. Tissue sections were co-stained with anti-CD163 to locate CD163+ TAMs. Data represent 4 different metastatic melanoma cases. Scale bars represent 100 µm. Magnification: x20 **(D)**. ****P <0.0001, *** P < 0.001, ** P < 0.01.

Thus, monocytes isolated from human blood were treated with various cytokines, and their proliferation was subsequently tracked. In contrast to an earlier observation (20), monocytes did not proliferate in response to the FMS-like tyrosine kinase 3 ligand (FLT3L) alone (**Figure 3A**). However, a proportion of monocytes divided in response to M-CSF or IL-3 (**Figure 3A, B**, **Supplementary Figure 2**). IL-3 synergised with M-CSF and induced robust proliferation of a subset of monocytes obtained from multiple donors (**Figures 3A, B**). Monocytic cells treated with IL3 or the combination of IL-3 and M-CSF highly expressed PD-L1 but expressed lower levels of HLA-DR and CD14 compared to those treated with M-CSF only (**Figure 3C**), consistent with the phenotype of CD163+ TAMs. Therefore, IL-3 efficiently induces the proliferation of monocytes, enhanced by M-CSF, but downmodulates HLA-DR expression, suggesting a potentially immunoregulatory phenotype. It has previously been reported that IL-3 is expressed in the vast majority of primary melanomas (27). When we probed for M-CSF transcripts in melanoma tissue, we found these are abundantly available in the melanoma TME, often in close proximity to CD163+ TAMs (**Figure 3D**).

Collectively, our data suggest that TME-derived signals, including IL-3 and M-CSF, induce CD163+ TAMs to proliferate within tumours as they differentiate from their monocytic precursors. This finding implies that human cancers may accumulate CD163+ TAMs within the TME not only by recruiting monocytes but also by driving the proliferation of those cells *in situ*.

### Monocytic cells treated with IL-3 and M-CSF modulate T cell proliferation

Next, we examined the functional properties of IL-3 + M-CSF-treated monocytic cells that resemble the proliferating CD163+ TAMs we observed in the TME. These IL-3 + M-CSF-treated “moTAMs” were compared with GM-CSF + IL-4-treated monocyte-derived dendritic cells (“moDCs”), and M-CSF-treated monocyte-derived macrophages (“moMACs”).

**Figure 4:**
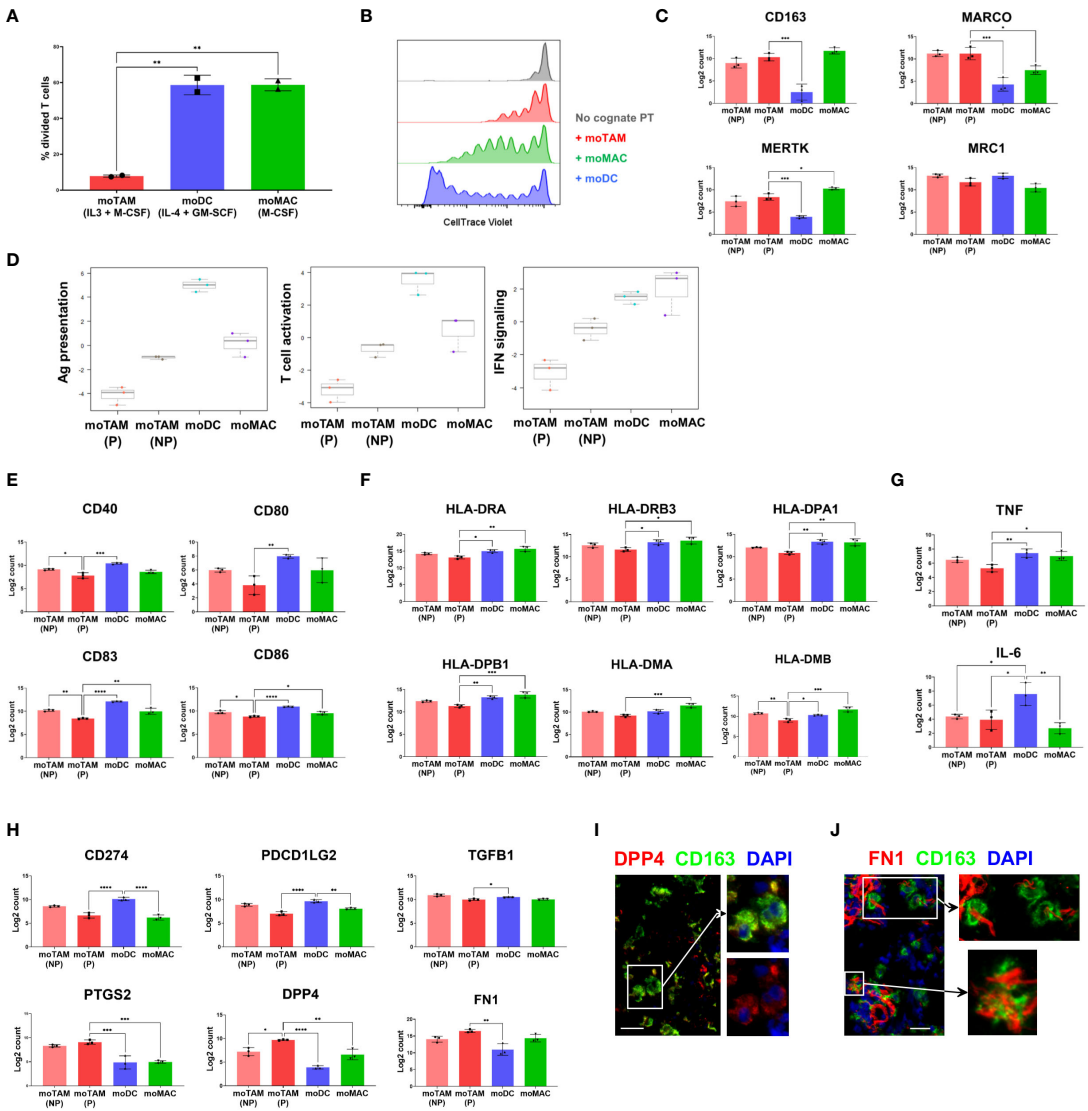
Functional and molecular characteristics of different monocytic populations. **(A)** CTV-labelled allogenic T cells were co-cultured with *in-vitro* generated moTAMs (IL-3 + M-CSF), moDCs (IL-4 + GM-CSF), or moMAC (M-CSF). The percentages of divided T cells, assessed by CTV dilution assay using flow cytometry, are shown. From live, single cells, co-cultured CD14+ monocytic cells were excluded, and T cells were identified as CD3+ cells. Data are representative of 4 independent experiments and shown as the mean with the error bars indicating the range of responses for duplicate samples. **(B)** Antigen-specific CD8+ T cell clones were labelled with CTV and added to PBMCs pulsed with ELA peptides. The cells were then co-cultured with moTAMs, moDCs or moMACs, and the division of T cell clones was measured. Grey indicates a negative control with the T cell clones cultured without the peptide-pulsed PBMCs. The results shown are from a replicate using untouched CD14+ monocytic cells cultured with different cytokines and representative of 4 different experiments. **(C)** Expression of the TAM-signature genes in monocytic cell subsets generated *in vitro*. **(D)** Pathway scores for Ag presentation, T cell activation, and IFN signalling were generated based on the gene expression profiles of different monocytic types. **(E–H)** Expressions of the genes encoding the activation markers **(E)**, MHC II molecules **(F)**, pro-inflammatory cytokines **(G)**, and immunosuppressive **(H)** molecules are shown. Bar graphs are from the experiment using 3 different monocyte donors and are shown as the mean with standard deviation. Statistically significant differences between different cell subsets are indicated (ANOVA): ****P <0.0001, *** P < 0.001, ** P < 0.01, *P < 0.05. **(I, J)** Protein expression of DPP4 and FN1 by CD163+ TAMs was examined by fluorescence microscopy. Data are representative of 3 different metastatic melanoma cases. Scale bars represent 25 µm. Magnification: x20 **(I, J)**.

We first assessed the ability of different monocytic cell types to modulate T cell proliferation post-activation by adding them to allogenic T cells (**Figure 4A**, **Supplementary Figure 3**). T cells cultured with moDCs or moMACs showed robust cell division in the presence of these allogenic monocytic cells (**Figure 4A**). In contrast, the proliferation of T cells was significantly impaired in the presence of moTAMs (**Figure 4A**), indicating the reduced capacity of moTAMs to activate T cells. Next, we compared the ability of these different monocytic populations to inhibit peptide:MHC-mediated expansion of antigen-specific CD8+ T cells. Clonal CD8+ T cells specific for MART1/Melan A (the HLA-A2-restricted peptide EAAGIGLTV) were labelled to allow their proliferation to be tracked by flow cytometry. They were then stimulated by HLA-A2+ PBMC, loaded with cognate peptide (the potent analogue peptide ELAGIGLTV) and co-cultured with each of these monocytic populations. Polarised monocytic populations were not loaded with cognate peptide and interacted with the activated clonal cells in a bystander capacity. In this setting, the co-culture with moDCs allowed the maximal expansion of clonal T cells (**Figure 4B**, **Supplementary Figure 4**). Although less efficient, clonal T cells co-cultured with moMACs could also undergo many rounds of division. In contrast, expansion and division of clonal T cells co-cultured with moTAMs was notably reduced (**Figure 4B**, **Supplementary Figure 4**), suggesting that when present in a bystander capacity these cells do not facilitate a microenvironment permissive of maximal T cell proliferation. Initial activation of the clonal T cells, as measured by CD137 upregulation 40h after peptide-loaded PBMC exposure, was equivalent across all conditions (**Supplementary Figure 5**), suggesting that the observed constraint on clonal T cell division was mediated post-activation, during the T cell proliferative cascade.

We then examined the gene expression profiles of moTAMs generated with IL-3 + M-CSF to better understand the molecular characteristics associated with their immunosuppressive features. Since this cytokine combination induces the proliferation of moTAMs *in vitro*, we first separated the proliferating fraction of moTAMs from the non-proliferating fraction. Subsequently, we compared their gene expression profiles with those of moDCs and moMACs by analysing RNA extracted from each cell type using a gene expression assay designed to target a range of myeloid-related genes. Four groups of *in vitro* generated monocytic cell types showed distinct gene expression profiles, and proliferating moTAMs showed similarities and differences to non-proliferating moTAMs (**Figure 4C–H**). moTAMs generated *in vitro*, including the proliferating fraction, expressed many genes that correlate with the molecular signatures of TAMs observed *in vivo*, including *CD163, MARCO, MERTK, and MRC1* (7) (**Figure 4C**). In line with their lesser capacity to enable T cell activity (**Figures 4A, B**), moTAMs had the lowest gene expression scores for antigen presentation, T cell activation, and interferon signalling (**Figure 4D**). In particular, compared to other cell types, proliferating moTAMs were marked by their lower expression of activation marker genes and MHC class II molecule genes (**Figures 4E, F**). Furthermore, moTAMs expressed much lower levels of the pro-inflammatory cytokine genes *TNF* and *IL-6* than moDCs (**Figure 4G**).

Consistent with the phenotype of CD163+ TAMs in the melanoma TME, moTAMs expressed the genes involved in immune suppression, including *CD274* (PD-L1), *PDCD1LG2* (PD-L2), and *TGFB1* (**Figure 4H**). However, the expression of *CD274* and *PDCD1LG2* was higher in moDCs than in moTAMs. Interestingly, the expression of several genes involved in immune regulation, namely *PTGS2* (encoding COX2), *DPP4*, and *FN1*, was much higher in proliferating moTAMs than in other cell types (**Figure 4H**). *DPP4* is implicated in immunoregulation and the fibrotic response (28), and FN1 plays a critical role in the TME during malignant transformation and metastasis (29). In some metastatic melanoma samples, we also observed a subset of CD163+ TAMs in melanoma tumours co-localised with DPP4, FN1, and COX2 proteins (**Figures 1G**, **4I, J**).

These results highlight the similarities between moTAMs generated *in vitro* and CD163+ TAMs observed *ex vivo*. The effects of IL-3 and M-CSF induce the proliferation of monocytic cells and skew their differentiation toward an immunosuppressive phenotype. Therefore, moTAMs provide a good model for studying the expansion of TAMs and their potential functions in tumours.

### IL-4 blocks the proliferation of monocytic cells induced by IL-3 and M-CSF

The different monocytic cell populations generated in various cytokine conditions showed marked differences in their molecular profiles, proliferative capacity, and ability to activate or suppress T cells. In particular, the gene expression data showed that moDCs generated with IL-4 and GM-CSF are more potent in antigen presentation and T cell activation than moTAMs and moMACs. We questioned whether IL-4, the cytokine capable of reversing a poor antigen-presenting cell function (30), affects the proliferation of moTAMs induced by IL-3 + M-CSF.

**Figure 5:**
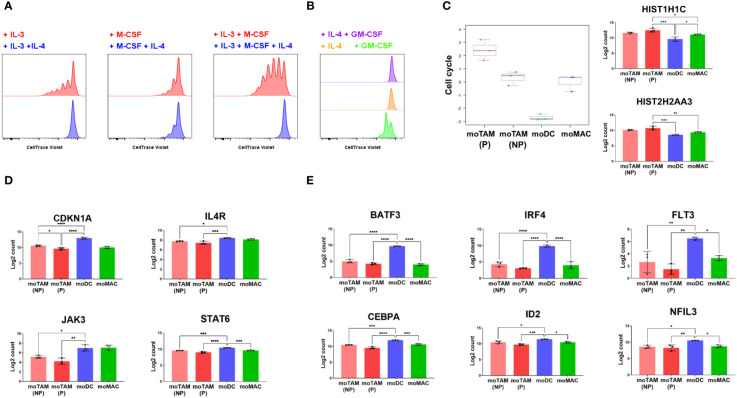
Antiproliferative effects of IL-4 on the proliferation of monocytic cells. **(A, B)** CTV-labelled monocytic cells were cultured in the indicated cytokine conditions, and their proliferation was tracked by flow cytometry. Data represent three independent experiments. **(C)** Bar graphs show cell cycle scores from *in vitro* generated monocytic cells and their expression of the genes involved in the cell cycle progression. **(D)** Bar graphs show the expression of CDKN1A, IL4R, JAK3 and STAT6 in different monocytic subsets. **(E)** Results show the relative expression of the genes involved in DC differentiation. Gene expression profiles shown in **(C–E)** were assessed by analysing RNA extracted from each cell type using the Human Myeloid Panel gene expression assay. Bar graphs above are from the experiment using 3 different monocyte donors and are shown as the mean with standard deviation. Statistically significant differences between different monocytic subsets are indicated (ANOVA): ****P <0.0001, *** P < 0.001, ** P < 0.01, *P < 0.05.

The addition of IL-4 completely blocked the proliferation of monocytic cells induced by IL-3 and M-CSF or IL-3 alone (**Figure 5A**). IL-4 also moderately inhibited the proliferation induced by treatment with M-CSF alone (**Figure 5A**). Furthermore, while GM-CSF alone induced some detectable proliferation, moDCs generated with both IL-4 and GM-CSF and those cultured with IL-4 only showed no cellular divisions (**Figure 5B**), consistent with the antiproliferative effects of IL-4. The gene expression analysis confirmed that moDCs downmodulate the genes involved in the cell cycle progression, whereas moTAMs highly express these genes (**Figure 5C**).

Our results also revealed that the expression of *CDKN1A*, the gene that encodes the potent cyclin-dependent kinase inhibitor p21^cip1/waf1^, was much higher in moDCs compared to moTAMs and moMACs (**Figure 5D**). Several genes involved in IL-4 signalling, such as *IL4R*, *JAK3*, and *STAT6*, were higher in moDCs (**Figure 5D**). The induction of *CDKN1A* in moDC correlated with the expression of the genes involved in the development of DCs (31), which include *BATF3*, *IRF4*, FLT3, *CEBPA*, *ID2*, and *NFIL3* (**Figure 5E**). These results suggest that as IL-4 drives monocyte-derived cells to acquire a more potent antigen-presenting cell type phenotype, it also blocks proliferative potential by inducing expression of p21^cip1/waf1^. In contrast, the differentiation toward moTAMs is associated with an immunosuppressive phenotype and a higher potential to proliferate, in the absence of p21^cip1/waf1^.

## Discussion

TAMs represent an emerging target for cancer therapies since they create an immunosuppressive TME and promote tumour metastasis. Their pro-tumoural activities also limit the efficacy of chemotherapy, anti-angiogenic therapy, radiotherapy and immunotherapy (32). Therefore, numerous pre-clinical studies and clinical trials have attempted to deplete TAMs in combination with other treatments.

CD163 is one of the most widely used markers to identify TAMs. In melanoma, the accumulation of CD163+ TAMs is associated with poor outcomes (4, 13, 33). Our results showed that CD163+ TAMs frequently infiltrated dermal and LN melanoma metastases, where they closely interact with both melanoma tumour and T cells. CD163+ TAMs expressed multiple molecules involved in immune suppression, including PD-L1, PD-L2, IL-10 and TGF-β1. An earlier report noted the correlation between CD163 and COX2 in melanoma (4), and we indeed confirmed the COX-2 expression in a subset of CD163+ TAMs. PGE2, a direct product of COX-2, is present at high levels in melanoma and is involved in suppressing the infiltration, proliferation, and activities of T cells (34, 35). Some CD163+ TAMs also expressed *FN1* and *DPP4*, exemplifying their potential role in regulating ECM and immune activities (28, 29).

Despite their potential roles in tumour progression, the origin of CD163+ TAMs in human melanoma has not been thoroughly investigated. While CD163 expression on a subset of human monocytes has previously been noted, recent studies have also identified a DC population expressing CD163 (36–38). It was also unclear whether monocyte-derived cells proliferate within melanoma tumours and subsequently give rise to TAMs, as demonstrated in mice.

Our data demonstrated that CD163+ TAMs accumulated in human metastatic melanoma display molecular phenotypes that strongly suggest they are derived from CD14+ CCR2+ monocytes. Our results are consistent with the recent single-cell study suggesting that human TAMs often share transcriptional signatures with circulating human monocytes (2). At the protein level, our imaging data suggest the transition from CD14 ^hi^ CCR2 ^hi^ monocytes to CD163 ^hi^ CD14 ^lo^ CCR2 ^lo^ TAMs i*n situ*. Earlier findings in mice identified the key role of CCL2 in recruiting CCR2+ monocytes to metastatic sites, where they promote the extravasation of tumour cells and facilitate tumour metastasis (39). We also detected CCL2 in melanoma tumours, consistent with an earlier observation (40). Therefore, CCL2 will likely be crucial in recruiting the CCR2+ monocytic precursors of CD163+ TAMs to tumours, as reported for CCR2+ MDSCs (41). It is plausible that other monocyte-recruiting chemokines are also involved in this process. In this regard, we confirmed the presence of CX3CL1 in melanoma tumours. CX3CL1 expression was also detected in other skin cancer types, along with the infiltration of CX3CR1+ macrophages (42). The CD16− and CD16+ subsets of human monocytes show differential expression of CCR2 and CX3CR1, and the CD16+ subset is higher in CX3CR1 but lower in CCR2 (43). We did observe some expression of CD16 in CD163+ TAMs, so we cannot rule out the possibility that some of the CD163+ TAMs that co-express CD16 derive from the CD16+ (non-classical & intermediate) monocytes recruited into the tumour. Further studies are required to establish the relative contribution of the CCL2-CCR2 and CX3CL1-CX3CR1 axis in the recruitment of the precursors of CD163+ TAMs.

Our results also showed that a subpopulation of CD163+ TAMs proliferates within the TME of melanoma, lung cancer and breast cancer. The concept that monocyte-derived TAMs can proliferate is supported by a study in a murine tumour model (8). Consistent with our findings, a recent single-cell analysis showed the accumulation of proliferating macrophages in the tumours from lung, colon, breast, stomach, pancreas, and liver cancer patients (2). In the publicly available data provided by the aforementioned study, we noted *CD163* expression in the proliferating macrophage subset, confirming CD163+ TAMs proliferate in multiple cancer types. Interestingly, this proliferating macrophage subset (2) expresses a range of other TAM-associated genes, including *SPP1*, *TREM2* and *FN1*. SPP1+ TAMs are thought to promote tumour growth and invasion by regulating ECM (44). TREM2+ TAMs represent a highly immunosuppressive TAM subset associated with T cell exhaustion (45). FN1 secreted by TAMs is thought to facilitate tumour progression (46). Therefore, the concept that monocytes recruited into tumours differentiate into proliferating CD163+ TAMs with immunosuppressive properties concurs with both published murine data and recent single-cell data from human tumours.

While it was previously believed that monocytes had limited proliferative capacity, we have demonstrated *in vitro* that human monocytes can proliferate robustly in response to the myelopoietic cytokines available in the TME. In particular, the combination of M-CSF and IL-3 proved to be remarkably effective in inducing a surprising level of proliferation in a subset of monocytic cells. We showed that M-CSF transcripts are abundantly present in the melanoma TME, often closely associated with the CD163+ TAMs. A mouse tumour model also showed that M-CSF induces the proliferation and differentiation of monocytes into TAMs, especially MHCII ^low^ TAMs (47). An earlier study has reported that M-CSF is produced in human melanoma tumours and elevated in the circulation of patients, and also correlates with the presence of CD163+ cells in the TME (33). Interestingly melanoma cell lines produced M-CSF when exposed to CD8+ T cells, suggesting its expression may be induced by CD8+ T cell attack (33). Yet while M-CSF in patients also correlates with the degree of CD8+ T cell infiltration in the TME, it paradoxically correlates to a lack of response to therapy with anti-PD-1 (33). We therefore speculate that M-CSF-driven proliferation of CD163+ TAMs may be potentiated by T cell attack on melanoma cells, leading to increased expression of PD-L1, PD-L2 and other immune-suppressive molecules in the TME. This provides a plausible mechanism for acquired resistance to anti-PD-1 therapy in patients in the presence of a robust CD8+ T cell response.

IL-3 is a pro-tumoural cytokine reportedly present in more than 80% of primary malignant melanoma (27). Both human and murine melanoma cells produce IL-3 (48, 49). IL-3 is also expressed by activated tumour-infiltrating lymphocytes, Tregs and tumour-derived endothelial cells (50–52). Furthermore, serum IL-3 is elevated in various cancers, including colorectal, pancreatic and non-small cell lung cancer (53–55). Our data demonstrated that proliferating moTAMs generated with IL-3 in combination with M-CSF resemble the CD163+ TAMs found in the melanoma TME. These proliferating moTAMs express not only *CD163* but also other well-known TAM-related genes (e.g. *MARCO, MERTK*) and the genes associated with immune suppression (e.g. *TGFB1, PTGS2*) and ECM modulation (e.g. *FN1, DPP4*). In addition, proliferating moTAMs were marked by their low expression of the genes involved in antigen presentation and T cell activation. Compared to other monocyte-derived cell types including moDCs and moMACs, moTAMs generated with IL-3 and M-CSF were superior in modulating the proliferation of allogeneic T cells and CD8+ T cells specific for an immunodominant melanoma antigen. These results support the idea that TME-derived signals induce monocytic precursor cells to undergo *in situ* proliferation to increase cell numbers, and subpopulations of the proliferating cells subsequently differentiate into the CD163+ TAMs that poorly support anti-tumour T cell activities *in situ*.

Interestingly, our data revealed that IL-4 completely blocks the proliferation of monocytic cells induced by IL-3 or the combination of IL-3 and M-CSF. Furthermore, monocytic cells treated with IL-4 or the combination of IL-4 and GM-CSF showed no proliferation. The latter cells were the canonical monocyte-derived DCs and were unique amongst our monocyte-derived cell subtypes in expressing classical DC lineage genes such as *BATF3 and IRF4*, consistent with their high potency in stimulating T cell proliferation. These moDCs did not proliferate and had strongly upregulated *CDKN1A*, implicating the cell cycle control protein p21^cip1/waf1^ in IL-4-driven cell cycle arrest. Previous animal studies reported contradictory results on the role of IL-4 in inducing (56, 57) or suppressing (58) the expansion of macrophage and monocyte-derived populations. Our results are consistent with the latter, in particular, the finding that IL-4 inhibits the proliferation of murine bone marrow-derived macrophages through *CDKN1A* induction via the JAK3-STAT6 pathway (58). Hence, we conclude that proliferation of human monocytic cells driven by IL-3 with or without M-CSF can be arrested by IL-4, most likely by increasing intracellular concentrations of p21^cip1/waf1^.

Our findings suggest potential strategies to block the accumulation of CD163+ TAMs. Targeting the CCR2-CCL2 axis will likely hamper the recruitment of monocytic TAM precursors into tumours (1). Neutralising the activities of TME-derived myelopoiesis cytokines (e.g. M-CSF, IL-3) using antibodies and small molecules might be beneficial in reducing the local expansion of TAMs. Another intriguing possibility is to utilise IL-4 to inhibit the proliferation of TAMs before re-educating them towards a more immune-supportive phenotype (59). While IL-4 has been proposed to be the primary activator of pro-tumoural TAMs such as the “M2” phenotype (60), it also has some anti-tumour effects thought to be related to the maturation of myeloid precursor cells (61). Other paradoxical effects of IL-4 on antigen presenting cells have been reported, such as enhancing IL-12 production, perhaps as part of negative feedback loops (62). The effects of IL-4 on monocytic cells in the TME are therefore much subtler than suggested by the simplistic “M1/M2” dichotomy, which poorly reflects the full diversity of TAMs (2, 8). Therefore, although using IL-4 to improve tumour immunity may initially seem counter-intuitive, it may be worth testing experimentally whether IL-4 can arrest proliferation of immunosuppressive TAMs and improve anti-tumour immune activity.

In conclusion, this study extends our knowledge of the origin of CD163+ TAMs and provides therapeutic insight for targeting them for cancer treatment. Further studies, particularly those using single-cell transcriptomics, will lead us to explore the heterogeneity within the CD163+ TAMs and help design innovative and more specific immunotherapy strategies.

## Data availability statement

The original contributions presented in the study are included in the article/**Supplementary Material**. Further inquiries can be directed to the corresponding author.

## Ethics statement

The studies involving humans were approved by Northern Regional Ethics Committee, New Zealand. The studies were conducted in accordance with the local legislation and institutional requirements. The participants provided their written informed consent to participate in this study.

## Author contributions

SP: Conceptualization, Data curation, Formal analysis, Investigation, Methodology, Validation, Visualization, Writing – original draft, Writing – review & editing. C-JC: Data curation, Writing – review & editing, Investigation. DV: Data curation, Formal analysis, Investigation, Methodology, Visualization, Writing – original draft, Writing – review & editing. MO: Data curation, Writing – review & editing. AB: Methodology, Writing – review & editing. RM: Resources, Writing – review & editing. JM: Resources, Writing – review & editing. PE: Resources, Writing – review & editing. PD: Conceptualization, Formal analysis, Funding acquisition, Methodology, Project administration, Resources, Supervision, Writing – original draft, Writing – review & editing.
