# Supplementary material for: Proliferating macrophages in human tumours show characteristics of monocytes responding to myelopoietic growth factors
Source: Front Immunol. 2024 Jun 5;15:1412076. doi: 10.3389/fimmu.2024.1412076 (PMC11188303; doi:10.3389/fimmu.2024.1412076)
Supplement: Supplementary file 1 [file DataSheet_1.pdf]

Supplementary Material

Supplementary Table 1

Metastatic site	Disease Stage	Site of metastasectomy	Extent of LN involvement	Known Mutations, Phenotypes	Checkpoint inhibitor therapy prior or after OT	Clinical response
**LN metastasis**						
1	III	Neck	NA	NA	NO IMMUNE THERAPY	STABLE DISEASE
2	III	R) Axilla	4/19 Positive	BRAF NEG	NO IMMUNE THERAPY	PROGRESSIVE DISEASE
3	III	L) Axilla	4/28 Positive<br>1/25 from Axillary	NA	NO IMMUNE THERAPY	NO EVIDENCE OF RECURRENCE
4	III	R) Neck	4/57 Positive	NA	NO IMMUNE THERAPY	NO EVIDENCE OF RECURRENCE
5	III	L) Axillar and lungs	11/23 Positive	BRAF NEG	NO IMMUNE THERAPY	DECEASED
6	IV	L) Axilla	1/34 Positive	BRAF NEG	NO IMMUNE THERAPY	METASTASIZED TO BRAIN
7	IV	R) Axilla	1/33 Positive	BRAF NEG	CHECKMATE TRIAL. PROGRESSIVE DISEASE ON TRIAL AND CAME OFF STUDY	PROGRESSIVE DISEASE
8	III	L) Axilla	4/21 Positive	NA	NO IMMUNE THERAPY	NO EVIDENCE OF RECURRENCE
9	IV	R) Axilla L) Neck	1/17 Positive from Neck. 0/31 Axilla	BRAF WILD TYPE	NO IMMUNE THERAPY	STABLE DISEASE
10	III AT PXN THEN IV	Left Groin	3/6 Positive. INOPERABLE PELVIC NODES	BRAF POS	DABRAFENIC/COBIMETINIB	DECEASED
11	IV	R) Neck, L) Axilla	NA	NA	NO IMMUNE THERAPY	NO NEW DISEASE
**Dermal metastasis**						
1	IV	right deltoid	NA	BRAFV600E	Dabrafenib & trametinib preop	PROGRESSIVE DISEASE
2	IV	left posterior neck	NA	BRAFV600E	Dabrafenib & trametinib preop	PROGRESSIVE DISEASE
3	IIIC	right heel	8/10 Positive	none detected	NO IMMUNE THERAPY	DECEASED
4	III	right pinna	NA	NA	NO IMMUNE THERAPY	Complete response to surgery
5	IIIC	right lower leg	NA	BRAF POS (subtype K601E)	NO IMMUNE THERAPY	Durable response to surgery
6	IV	left shoulder	NA	NA	NO IMMUNE THERAPY	Distant recurrent disease

Clinical features of metastatic melanoma assessed in this study. NA: information not available. ND: Not determined

Supplementary Table 2

	Disease Stage	Primary or metastatic	Extent of LN involvement	Known Mutations, Phenotypes
**Lung adenocarcinoma**				
1	IV	Primary		EGFR mutation
2	IV	Primary		EGFR mutation
3	IV	LN metastasis	2/07 positive	
4	IV	LN metastasis	6/6 positive	
5	IV	Visceral metastasis (liver)		
6	IV	Bone (Humerus)		
**Breast cancer**				
1	IV	Primary		HER2 +
2	IV	Primary		HER2 -
3	IV	Primary		HER2 -
4	IV	Primary		HER2 -
5	IV	Primary		HER2 +
6	IV	Primary		HER2 +
7	IV	Primary		HER2 -
8	IV	Primary		HER2 -
9	IV	LN metastasis	1/18 nodes positive	HER2 +
10	IV	LN metastasis	7/8 nodes positive	HER2 -
11	IV	LN metastasis	4/4 nodes positive	HER2 -
12	IV	LN metastasis	6/7 nodes positive	HER2 -
13	IV	LN metastasis	4/8 nodes positive	HER2 +
14	IV	LN metastasis	3/78 nodes positive	HER2 +
15	IV	LN metastasis	1/12 nodes positive	HER2 -
16	IV	LN metastasis	6/8 nodes positive	HER2 -
17	IV	Visceral metastasis (lung)		HER2 +
18	IV	Visceral metastasis (lung)		HER2 -
19	IV	Visceral metastasis (liver)		HER2 -
20	IV	Visceral metastasis (liver)		HER2 +
21	IV	Bone (femur)		HER2 -
22	IV	Visceral metastasis (lung)		HER2 -

Clinical features of lung and breast cancer assessed in this study. NA: information not available

Supplementary Table 3

Antibody (Ab)	Species	Clone	Cat #	Manufacturer
Immunofluorescence Ab				
**CCR2**	Rabbit	E68	ab32144	Abcam
**CD3**	Rabbit	MRQ-39	103R-95	Cell Marque
**CD3**	Mouse	UCHT1	555330	BD Biosciences
**CD14**	Mouse	7	ab49755	Abcam
**CD16**	Rabbit	SP175	116R-14	Cell Marque
**CD68**	Mouse	Y1/82A	333802	BioLegend
**CD163**	Mouse	EDHu-1	MCA1853	Serotec
**CD163**	Mouse	3B4	LS-B10966	LSBio
**COX-2**	Rabbit	D5H5	12282T	Cell Signaling Technology
**DPP4**	Mouse	BA5b	302702	BioLegend
**Fibronectin**	Mouse	2B6-D4	555867	BD Biosciences
**HLA-DR**	Mouse	TU36	555559	BD Biosciences
**Ki-67**		SP6	275R-15	Cell Marque
**MART1 (Melan A)**	Mouse	M2-7C10	CMC755	Cell Marque
**PD-L1**	Rabbit	28-8	ab205921	Abcam
**PD-L2**	Mouse	MIH14	ab110182	Abcam
**Sox10**	Mouse	Sox10/991	ab212843	Abcam
Flow cytometry Ab				
**CD3 PE-Cy5**	Mouse	UCHT1	300410	BioLegend
**CD11c Alexa Fluor 700**	Mouse	Bu15	337219	BioLegend
**CD14 FITC**	Mouse	M5E2	301804	BioLegend
**HLA-DR BV605**	Mouse	L243	307640	BioLegend
**PD-L1 PE**	Mouse	29E.2A3	329706	BioLegend

List of antibodies used in this study.

Supplementary Figure 1

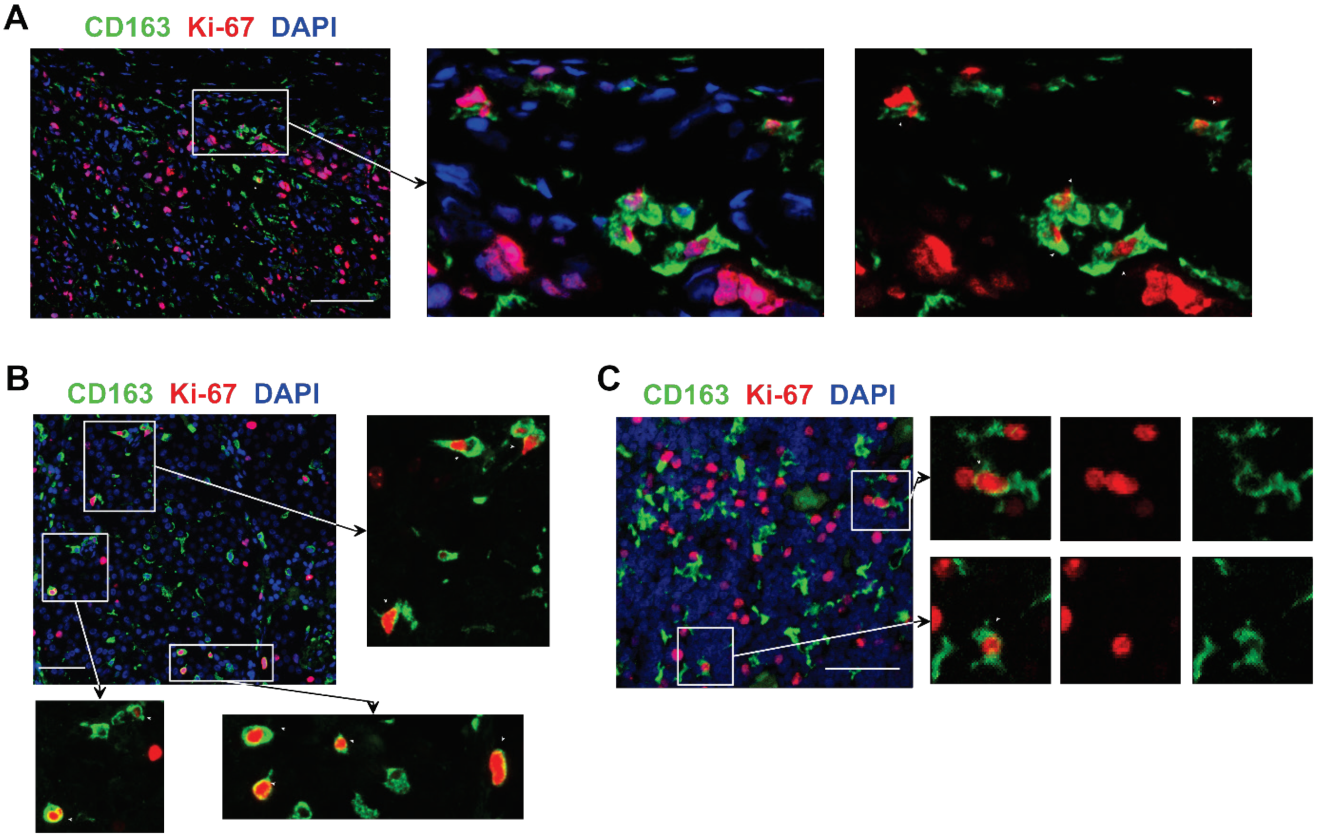

Proliferating CD163+ TAMs in multiple cancer types. (A-C) Tissue sections from metastatic melanoma (A), breast cancer (B) and lung cancer (C) were probed with antibodies against CD163 and Ki-67 to assess the proliferation of CD163+ TAMs. Results shown are representative of 15 melanoma, 8 breast, and 4 lung cancer cases. Scale bars represent 100 (A) or 50 (B-C) µm.

Supplementary Figure 2

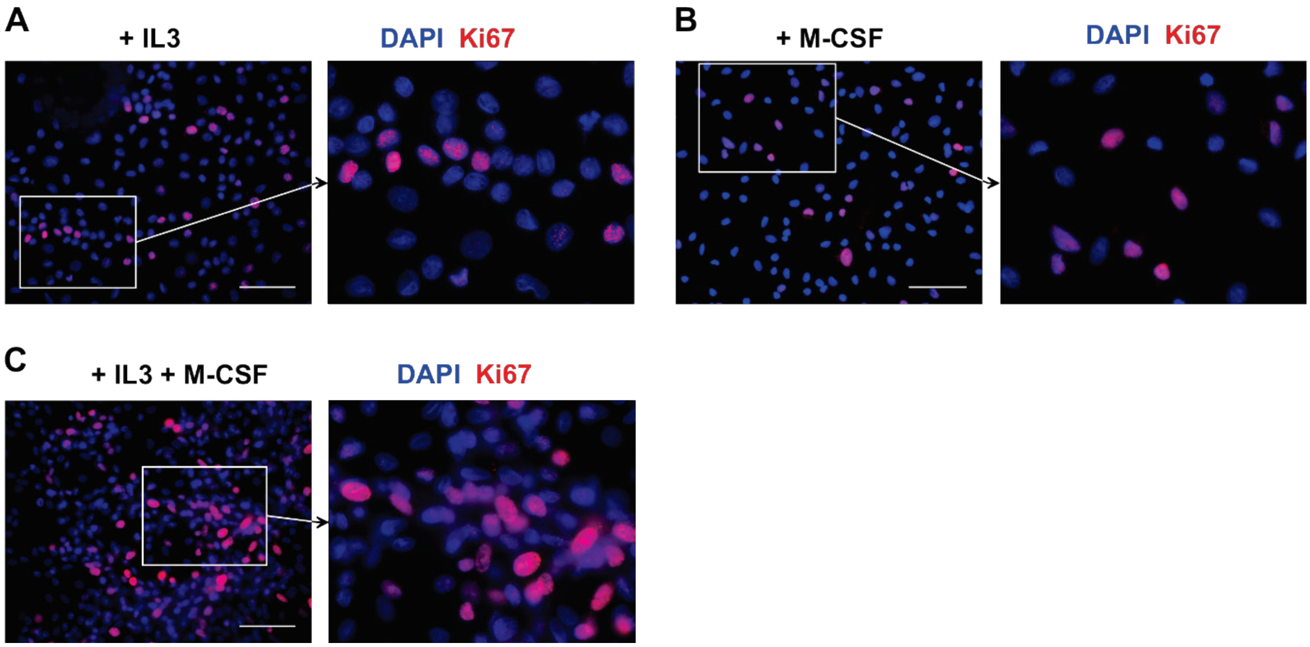

Proliferation of in vitro-generated human monocytic cells. Monocytic cells were generated using the indicated cytokines. Subsequently, cells were fixed and stained for Ki67 expression to assess their proliferation. Data are representative for 3 replicates. Scale bars represent 50 µm.

Supplementary Figure 3

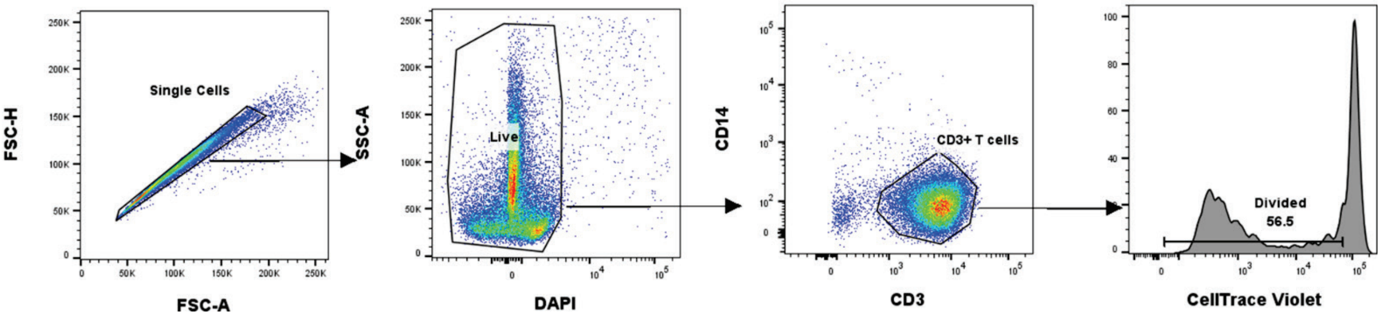

Representative flow cytometry plots and gating strategy. CTV-labelled allogenic T cells were co-cultured with *in-vitro* cultured monocytic cells. The percentages of divided T cells, assessed by CTV dilution assay using flow cytometry, are shown. Gating strategy to identify the T cells that underwent cell division is shown. From live, single cells, co-cultured CD14+ monocytic cells were excluded, and T cells were identified as CD3+ cells.

Supplementary Figure 4

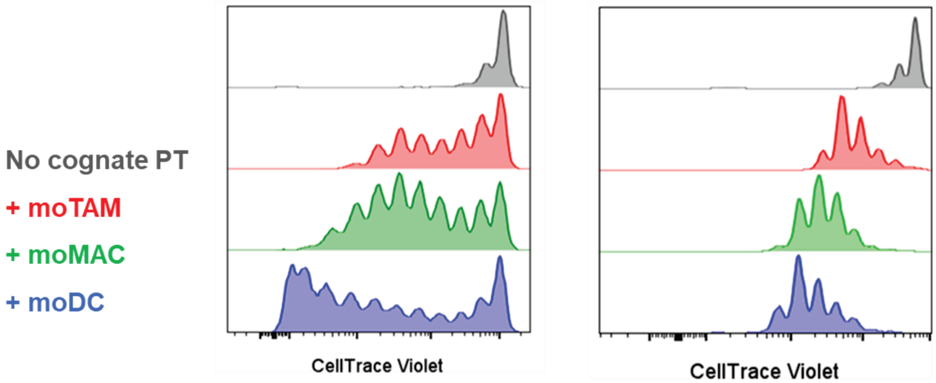

Inhibition of antigen-specific T cell proliferation by moTAM. Antigen-specific CD8+ T cell clones were labelled with CTV and added to PBMCs pulsed with ELA peptides. The cells were then co-cultured with moTAMs, moDCs or moMACs, and the division of T cell clones was measured. Grey indicates a negative control with the T cell clones cultured without the peptide-pulsed PBMCs. The results shown are from two experiments using CD14+ monocytic cells acquired from different donors by positive magnetic sorting and subsequently cultured with different cytokines.

Supplementary Figure 5

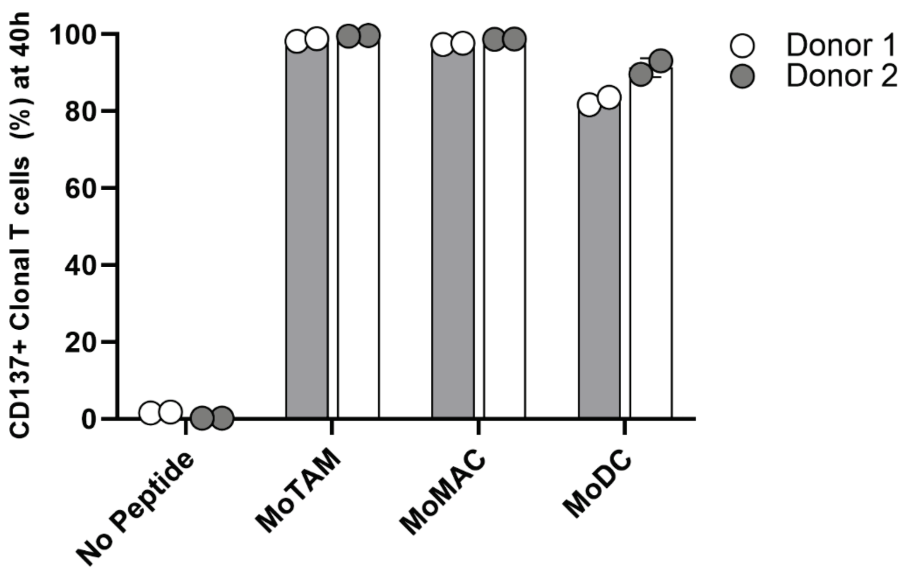

CD137 upregulation at 40 hr after exposure to peptide-loaded PBMC . Antigen-specific CD8+ T cell clones were labelled with CTV and added to PBMCs pulsed with ELA peptides. The cells were then co-cultured with moTAMs, moDCs or moMACs, and T cell activation was assessed by measuring CD137 expression. The results shown are from two experiments using CD14+ monocytic cells acquired from different donors by positive magnetic sorting and subsequently cultured with different cytokines.
